# High red blood cell composition in clots is associated with successful recanalization during intra-arterial thrombectomy

**DOI:** 10.1371/journal.pone.0197492

**Published:** 2018-05-21

**Authors:** Jong Wook Shin, Hye Seon Jeong, Hyon-Jo Kwon, Kyu Sang Song, Jei Kim

**Affiliations:** 1 Daejeon-Chungnam Regional Cerebrovascular Center, Chungnam National University Hospital, Daejeon, South Korea; 2 Department of Neurology, Hospital and School of Medicine, Chungnam National University, Daejeon, South Korea; 3 Department of Neurosurgery, Hospital and School of Medicine, Chungnam National University, Daejeon, South Korea; 4 Department of Pathology, Hospital and School of Medicine, Chungnam National University, Daejeon, South Korea; Maastricht University Medical Center, NETHERLANDS

## Abstract

We evaluated the composition of individual clots retrieved during intra-arterial thrombectomy in relation to recanalization success, stroke subtype, and the presence of clot signs on initial brain images. We analyzed clot and interventional data from 145 retrieval trials performed for 37 patients (69.5±14.0 years, 20 men, large artery atherosclerosis, n = 7; cardioembolism, n = 22; undetermined etiology, n = 8) who had undergone intra-arterial thrombectomy. Rates of clot retrieval and successful recanalization (Arterial Occlusive Lesion score of 2–3) for separate retrieval trials were evaluated. The area occupied by red blood cell (RBC), fibrin/platelets, and white blood cell (WBC) was measured from digitized images of hematoxylin-eosin stained clots. Compositional differences were compared according to recanalization success, stroke subtype, and the presence of hyperdense clot sign on initial computed tomography and/or blooming artifact on magnetic resonance image. Of the 145 total retrieval trials (3.4±2.4 times per patient), clot was retrieved in 93 trials (64%), while recanalization was successful in 73 (50%). Fibrin/platelets (63%) occupied the greatest area in retrieved clots, followed by RBCs (33%) and WBCs (4%). Clots retrieved from successful recanalization exhibited higher RBC composition (37%) than those retrieved from non-recanalization trials (20%, p = 0.001). RBC composition was higher in cardioembolic stroke (38%) rather than large artery atherosclerosis (23%) and undetermined etiology (26%, p = 0.01). Clots exhibiting clot signs (40%) had higher RBC composition than those without clot signs (19%, p = 0.001). RBC-rich clots were associated with successful recanalization of intra-arterial thrombectomy, cardioembolic stroke, and the presence of clot-signs on initial brain images.

## Introduction

Ischemic stroke is a serious condition with a high risk of lasting neurological disability. Recanalization after the occlusion of an intracranial artery is important to restoring cerebral perfusion below the occluded site. [1] In particular, successful recanalization within the time window is critical for improving the clinical outcome of acute ischemic stroke patients. [2]

Intra-arterial thrombectomy (IAT) is effective in ensuring reperfusion following occlusion of intracranial vessels in patients with acute ischemic stroke [3]. Rapid reperfusion of the occluded vessel and surrounding regions within 6 h of stroke onset has been regarded as critical for achieving good functional outcomes following IAT [1,2,4,5]. However, reperfusion within this time window is unsuccessful in approximately two-fifths of patients treated via IAT [4]. Increases in the interval between symptom onset and recanalization (e.g., late arrival at the emergency room (ER) following symptom onset and delay of IAT following admission) significantly increase the risk of unsuccessful reperfusion [6]. Additional studies have revealed that individual variations in vessel anatomy [7] and intracranial collateral development [8] may also contribute to delayed or unsuccessful recanalization.

Recently, several research groups have evaluated the association between the histological composition of clots retrieved after IAT and successful reperfusion among patients with various subtypes of stroke [9–13]. Such studies have examined the effect of red blood cell (RBC), fibrin/platelet, and white blood cell (WBC) composition within retrieved clots on rates of successful reperfusion using specific retrieval devices, as well as the influence of clot signs such as hyperdense vessels on initial computed tomography (CT) and blooming artifacts on gradient-echo magnetic resonance imaging (GRE MRI) [9–13]. However, in these previous studies, recanalization status was evaluated based on the final reperfusion status of the occluded vessels following the complete IAT intervention, rather than after each catheterization step performed during IAT [10–13]. IAT interventions typically require several catheterization steps to ensure complete reperfusion, as unsuccessful reperfusion occurs in some trials even after clot retrieval. Thus, the final reperfusion status obtained after multiple catheterizations is inappropriate for analyzing the association between clot composition and clot retrieval/successful recanalization, which should be evaluated after the individual catheterization steps during IAT.

The present study aimed to evaluate the association between the histological composition of retrieved clots and successful recanalization after separate retrieval trials during IAT. We also compared the composition of individual clots according to stroke subtypes, clot retrieval devices, and the presence of clot signs on initial brain images.

## Materials and methods

### Patients

A total of 46 patients who had undergone IAT for the treatment of acute ischemic stroke between February 2014 and May 2017 were enrolled in the present study. All patients met the following inclusion criteria: age >18 years, time from onset to groin puncture <6 h, National Institutes of Health Stroke Scale (NIHSS) score ≥4, occlusion of a unilateral middle cerebral artery (MCA) and/or terminal internal carotid artery (ICA) confirmed via cerebral angiography. For each patient, we collected data regarding demographic characteristics, history of cerebrovascular risk factors (e.g., hypertension, diabetes, smoking, atrial fibrillation, and heart failure), and initial stroke severity as determined by NIHSS scores. Stroke subtypes were classified in accordance with the Trial of ORG10172 in Acute Stroke Treatment (TOAST) [14] criteria, as follows: large artery atherosclerosis (LAA), cardioembolic stroke, stroke associated with undetermined causes. (S1 Table) The study protocol was approved by the Institutional Review Board of Chungnam National University Hospital (CNUH2014-10-015); the need for informed consent was waived due to the retrospective nature of the study.

### Intra-arterial thrombectomy (IAT)

IAT was performed as bridging therapy after intravenous thrombolysis (IVT), or as primary therapy in accordance with the critical pathway established at the Daejeon-Chungnam Regional Cerebrovascular Center of Chungnam National University Hospital [4]. Patients who arrived at the ER within 4.5 h of symptom onset with no hemorrhage or significant low-density lesions on initial CT images (i.e., less than one-third of the MCA territory) received 0.9 mg/kg of recombinant tissue plasminogen activator. Patients exhibiting no clinical improvement after IVT in addition to discrepancies between diffusion- and perfusion-weighted magnetic resonance images (MRIs) underwent IAT as bridging therapy. Following confirmation of the absence of hemorrhage on initial CT images, patients with discrepancies between diffusion-and perfusion-weighted MR images who arrived 4.5–6 h after symptom onset were treated via IAT. Retrieval devices for IAT were randomly chosen between a retrievable stent (Solitaire AB, ev3, Covidien, Dublin, Ireland) and a suction device (Penumbra System^®^, Penumbra Inc., Alameda, CA, USA) before the start of individual IAT interventions by an interventionist. (S1 Table)

### Evaluation of clot signs on initial brain images

We evaluated clot signs in occluded vessels on initial CT and/or MR images. On initial CT images, clot signs were defined based on hyperdensity of the MCA using the following criteria: spontaneous visibility of high density within the unilateral MCA, disappearance of bone windows, and absence of subarachnoid hemorrhage [15]. On initial gradient echo (GRE) MRI, hypointense signals exceeding the diameter of the contralateral MCA were defined as blooming artifacts suggesting a clot within occluded vessels [13]. (S1 Table)

### Evaluation of clot retrieval and recanalization after separate retrieval trials

Angiographic revascularization of occluded vessels requires two components: 1) recanalization of occluded vessels and 2) reperfusion past the occlusion and into the distal branches [16]. In the present study, we evaluated clot retrieval and recanalization status of the occluded vessel after separate retrieval trials using stent and/or suction devices. Clot retrieval was defined as visible clots captured inside or within the tip of the retrieval devices after each trial. Recanalization status was evaluated after each trial and classified as follows based on the Arterial Occlusive Lesion (AOL) recanalization score: 0, no recanalization of the primary occlusive lesion; 1, incomplete or partial recanalization with no distal flow; 2, incomplete or partial recanalization with any distal flow; 3, complete recanalization with any distal flow [17]. Successful recanalization was defined as an AOL score of 2 or 3, while unsuccessful recanalization was defined as an AOL score of 0 or 1 [18]. The final reperfusion status below the distal arterial beds of the initially occluded artery was determined after the final retrieval trial for each patient. The final reperfusion status was graded according to the Thrombolysis in Cerebral Infarction (TICI) scale, as follows: 0, no perfusion; 1, penetration with minimal perfusion; 2a, partial perfusion with only partial filling; 2b, partial perfusion with complete but slower distal filling; 3, complete perfusion [19]. The final successful reperfusion was defined when TICI grades 2b or 3 had been achieved [20]. (S1 Table)

Time intervals required to achieve the final successful reperfusion were evaluated from (1) symptom onset to groin puncture for IAT (onset-to-puncture time), (2) puncture to the final reperfusion (puncture-to-reperfusion time), and (3) symptom onset to the final reperfusion (onset-to-reperfusion time). (S1 Table) The number of total catheterizations, rate of clot retrieval, and/or rate of recanalization during the full IAT procedure were calculated based on stroke subtypes, occlusion locations, retrieval devices, and the final reperfusion status.

### Histopathological analysis of the retrieved thrombus

Clots retrieved after individual catheterizations were immediately fixed in 10% phosphate-buffered formalin. Formalin-fixed clots were embedded in paraffin and sliced (thickness: 4 μm) the center of the clot in 4 sections for hematoxylin and eosin (H&E) staining. To measure clot composition, one of the 4 H&E-stained clots were scanned using ScanScope CS (Leica Biosystems, Nussloch, Germany) at a magnification of 20x. The area of the clot occupied by RBCs, fibrin/platelets, and WBCs was determined using the Positive Pixel Count algorithm (ver. 9) of the ImageScope digital slide viewer (Ver 12.2, Leica Biosystems, Nussloch, Germany).

Prior to the measurement of clot composition, we set parameters for the hue, saturation, and intensity of the digitized clot images by following the guides of the Positive Pixel Count Algorithm (http://tmalab.jhmi.edu/aperiou/userguides/Image_Analysis_UG.pdf). The hue value for the digitized clot images was defined as 0.05 (red color) in the Positive Pixel Algorithm. To set the hue width for the digitized clot images, we compared the WBC segmentations in between 0.33, 0.4, and 0.5 of hue width (S1 Fig). Then, hue width was set at 0.4 to decrease over- and under-segmentation of WBC composition at the 0.05 hue value. To set saturation for the images, we compared the compositional differences of WBC by different saturation values from 0.01 to 0.1 in 6 clots having different compositions of RBC and fibrin/platelets (S2 and S3 Figs). The saturation value was set at 0.04 because no significant change of WBC compositions was observed from this value point (S2 and S3 Figs).

The Positive Pixel Count Algorithm uses three intensity values for strong positive (I_sp_), positive (I_p_), and weak positive (I_wp_) thresholds. I_sp_ was set at 100, following the guidelines of the algorithm. To set the I_wp_ threshold, we compared the compositional differences of RBC and fibrin/platelets from images digitized with different I_wp_ from 211 to 220 in the 6 different clots (S4 Fig). On the I_wp_ analysis, no significant compositional differences of RBC and fibrin/platelets were observed in the range of 211 to 220 of I_wp_ threshold. So, we set 215, the middle value between 211 and 220, as the I_wp_ threshold value for the present study. To set I_p_ values, we compared the compositional differences of RBC and fibrin/platelets from images digitized with different I_p_ values from 135 to 180 in 6 different retrieved clots (S5 Fig). On the I_p_ analysis, the RBC and fibrin/platelets compositions were different by the changes of I_p_ values in RBC-rich and mixed type of clots, even though the clot compositions were not significantly different in fibrin/platelets-rich clots by the change of I_p_ values. To measure the clot composition of a clot, an expert pathologist selected a best clot image of the 10 clot images differently digitized from 135 to 180 of Ip values.

**Fig 1:**
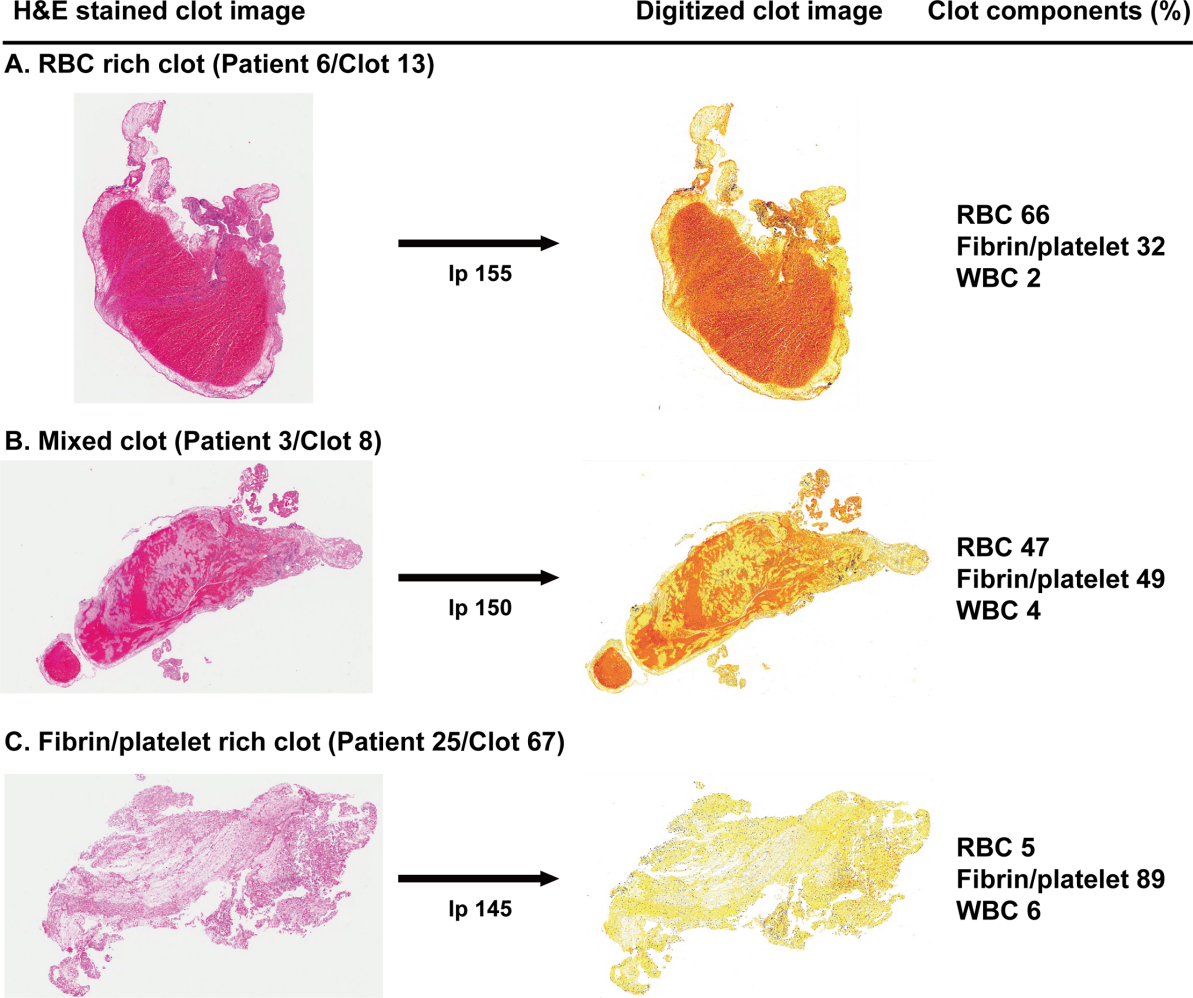
Measurement of RBC, fibrin/platelet, and WBC composition using the Positive Pixel Count (PPC) algorithm of the ImageScope program in an RBC-rich clot (A), a mixed clot (B), and a fibrin/platelet-rich clot (C). To measure the area of RBC and fibrin/platelet in a digitized clot image, hue value (0.05), hue width (0.4), saturation (0.04), the intensity of strong positive pixels (I_sp_, 100), and the intensity of weak positive pixels (I_wp_, 215) were set to the fixed values. The intensity of positive pixels (I_p_) was only adjusted in a best image of the 10 clot images differently digitized from 135 to 180 of I_p_ values to obtain the clearest discrimination of RBC and fibrin/platelet in the individual clot images.

The total values (in pixels) for the area occupied by the entire H&E-stained clot, RBCs (coded in red and orange), fibrin/platelets (coded in yellow), and WBCs (coded in blue) were measured from the adjusted clot images selected by a pathologist (Fig 1). The ratio of pixels representing RBCs, fibrin/platelets, or WBCs to the total number of pixels was calculated for each clot (S1 Table, Fig 1). We also evaluated the clot types by the enrichment of RBC or fibrin/platelet compositions according to the previous criteria [21]; 1) RBC-rich, which RBC outnumberd by >15% than fibrin/platelet area, 2) fibrin/platelets-rich, which fibrin/platelets outnumbered by >15% than RBC, 3) mixed, which both are not the case. (S1 Table)

### Statistical analysis

Baseline characteristics were summarized as means, frequency counts, or proportions. Cross-tab analysis was used to compare the total number of clots retrieved and rates of clot retrieval/successful recanalization according to stroke subtype, occlusion location, device, and the final reperfusion status. To test the compositional differences of retrieved clots between the stroke subtypes, we analyzed compositions of RBC, fibrin/platelet, and WBC in total patients as well as in total retrieved clots using analysis of variance (ANOVA). The compositional differences of the clots were also analyzed according to recanalization status after each retrieval trial, stroke subtype, the presence of clot signs, retrieval devices, and the performance of primary IVT before IAT using T-tests or ANOVA. To compared the clot types between stroke subtypes, we analyzed the frequency of RBC-rich, fibrin/platelet-rich, and mixed types in total retrieved clots between stroke subtypes using *χ*^*2*^-test. Statistical analysis was performed using SPSS (Ver. 22.0, SPSS Inc., Chicago). The level of statistical significance was set at p< 0.05.

## Results

Of the 46 initial patients who had undergone IAT, 37 patients (69.5±14 years old; 20 men) with intracranial artery occlusion (terminal ICA, n = 9; M1 portion of MCA, n = 28) were included in the final analysis of clot images. The remaining nine patients were excluded (extracranial ICA occlusion, n = 6; basilar artery occlusion, n = 1; insufficient clot sample, n = 2) (Table 1). The mean NIHSS score upon arrival at the ER was approximately 11.7±4.7, and 16 patients (43%) of the patients received IVT prior to IAT. The most frequent risk factors among patients include atrial fibrillation (62%), hypertension (54%), diabetes (24%), smoking (19%), and heart failure (16%). Cardioembolism (59%) was more frequent than LAA (19%) or stroke associated with undetermined causes (22%). Successful reperfusion (TICI grade of 2b or 3 at final reperfusion) was achieved in 31 (84%) of the 37 included patients. IAT was initiated within a mean of 210±72 minutes, and the final successful reperfusion was attained within a mean of 259±73 minutes after symptom onset (Table 1).

**Table 1:** Patient characteristics.

Characteristics	N = 37
Age (years, mean±SD)	69.5±14
Sex, Male	20(54.1%)
Risk Factors	
Atrial fibrillation	23 (62%)
Hypertension	20 (54%)
Diabetes	9 (24%)
Smoking	7 (19%)
Heart Failure	6 (16%)
TOAST classification	
Large Artery Atherosclerosis	7 (19%)
Cardioembolism	22 (59%)
Undetermined	8 (22%)
Occlusion location	
Terminal-ICA	9 (24%)
M1 of MCA	28 (76%)
Intravenous thrombolysis	16 (43%)
Initial NIHSS score (mean±SD)	11.7±4.7
Final successful reperfusion (TICI 2b and 3)	31 (84%)
Interventional time parameters (min, mean±SD)	
Onset to puncture	210 ±72
Puncture to final reperfusion	49 ± 42
Onset to final reperfusion	259±73

TOAST, Trial of ORG 10172 in Acute Stroke Treatment; ICA, intracranial artery; MCA, middle cerebral artery; NIHSS, National Institute of Health Stroke Scale; TICI, Thrombolysis in Cerebral Infarction scale

### Clot retrieval and recanalization following individual catheterization steps

Among the 37 included patients, clot retrieval was attempted a total of 145 times (3.4±2.4 times per patient) during IAT (Table 2). Of the total retrieval trials, 93 (64%, 2.5±1.3 times per patient) were associated with clot retrieval, and successful recanalization (AOL grade of 2–3) was ultimately achieved in 73 of the retrieval trials (50%, 1.9±1.1 times per patient). The rates of clot retrieval (LAA, 50%; cardioembolism, 66%; undetermined, 76%, p = 0.109) and successful recanalization (LAA, 34%; cardioembolism, 55%; undetermined, 56%, p = 0.220) did not significantly differ among stroke subtypes, although values for both were lowest in patients with LAA. Approximately two retrieval trials were required for clot retrieval and successful recanalization among patients with each of the three stroke subtypes. By the occlusion location, a significantly greater number of retrieval trials was required for retrieval of clots within the terminal ICA (5.8±2.0 times per patient) than for clot retrieval of those within the MCA (3.3±2.6 times per patient) (p = 0.015). However, rates of clot retrieval (terminal-ICA 60%; MCA, 67%) and successful recanalization (terminal ICA, 48%; MCA, 53%) were similar in both occlusion locations. No significant differences in rates of clot retrieval (stent, 61%; suction, 65%) or successful recanalization (stent, 44%; suction, 52%) were observed between the two device types. A significantly lower number of retrieval trials was required in patients who had achieved successful reperfusion (3.6±2.3 times per patient) than in those with unsuccessful reperfusion (5.7±4.0 times, p = 0.081). Rates for clot retrieval (TICI<2b, 56%; TICI = 2b or 3, 67%, p = 0.308) and successful recanalization (TICI<2b, 41.2%; TICI = 2b or 3, 53%, p = 0.434) were higher in patients who had achieved the final successful reperfusion, although this difference was not significant.

**Table 2:** Number of trials for clot retrieval and successful recanalization.

	Total retrieval trial no. (/pt.)	Clot retrieval		Successful recanalization	
		No. (/pt.)	Rate (/total no., %)	No. (/pt)	Rate (/total no., %)
Total patients (n = 37)	145 (3.4 ± 2.4)	93 (2.5 ± 1.3)	64	73 (1.9 ± 1.1)	50
Stroke subtypes					
LAA (n = 7)	32 (4.6 ± 4.1)	16 (2.3 ± 1.0)	50	11 (1.6 ± 1.3)	34
Cardioembolism (n = 22)	88 (4.0 ± 2.2)	58 (2.6 ±1.3)	66	48 (2.2 ± 1.1)	55
Undetermined (n = 8)	25 (3.1 ±2.7)	19 (2.4 ±1.8)	76	14 (1.8 ± 0.9)	56
Occlusion locations					
Terminal-ICA (n = 9)	52(5.8 ± 2.0)	31 (3.4 ± 0.9)	60	25 (2.8 ± 1.0)	48
MCA M1 (n = 28)	93 (3.3 ± 2.6)	62 (2.2 ±1.3)	67	48 (1.7 ± 1.0)	53
Retrieval devices					
Solitaire Stent	36	22	61	16	44
Penumbra Suction	109	71	65	57	52
The final reperfusion					
TICI < 2b (n = 6)	34 (5.7±4.0)	19 (3.2±1.2)	56	14 (2.3±1.6)	41
TICI 2b or 3 (n = 31)	111 (3.6±2.3)	74 (2.4±1.4)	67	59 (1.9±1.0)	53

LAA, large artery atherosclerosis; ICA, internal carotid artery; MCA, middle cerebral artery; TICI, Thrombolysis in Cerebral Infarction scale

### Composition of retrieved clots

**Fig 2:**
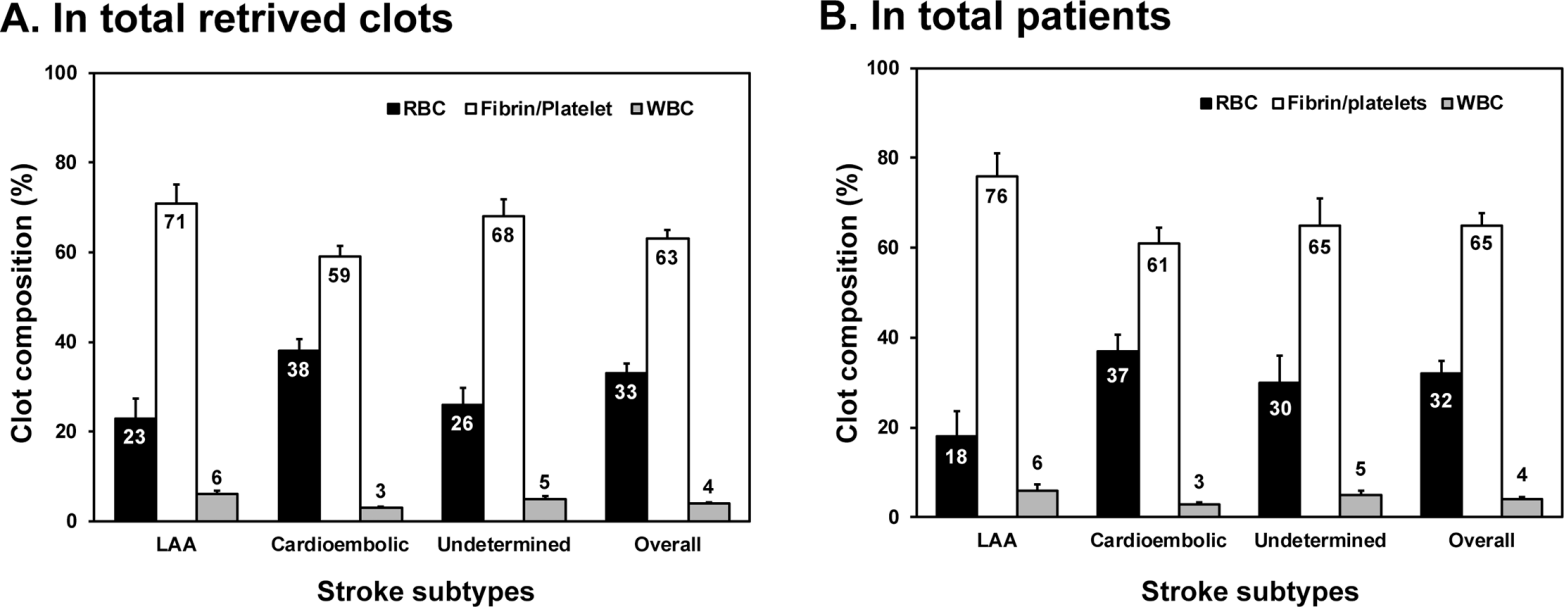
Comparison of clot composition (red blood cells [RBCs], fibrin/platelets, white blood cells [WBCs]) in total retrieved clots (A) and in total included patients according to stroke subtype. LAA: Large artery atherosclerosis.

We evaluated the overall composition of 93 retrieved clots. Fibrin/platelets (63%) represented the most prevalent composition of retrieved clots, followed by RBCs (33%) and WBCs (4%) (Fig 2A). The area occupied by RBCs was significantly greater in patients with cardioembolism than in patients with other types of stroke (LAA, 23%; cardioembolism, 38%; undetermined, 26%, p = 0.038). Accordingly, the area occupied by fibrin/platelets was lowest in patients with cardioembolism (LAA, 71%; cardioembolism, 59%; undetermined, 68%) (Fig 2A). Although WBCs represented a small fraction of the total clot area, WBC composition was lowest in patients with cardioembolism (LAA, 6%; cardioembolism, 3%; undetermined, 5%, p<0.001) (Fig 2A). On compositional analysis according to the stroke subtypes in all 37 patients, fibrin/platelets (65%) were more prevalent than RBCs (32%) and WBCs (4%) in the retrieved clots like as in the total clots analysis (Fig 2B). Cardioembolic patients showed significantly higher RBCs than other two subtypes of stroke (cardioembolism, 37%; LAA, 18%; undetermined, 30%, p = 0.05) as was the case in the total clot analysis. On the comparison of the clot types, fibrin/platelet-rich clot type was more frequent in LAA (81.3%) and undetermined (73.7%) subtypes than RBC-rich as well as mixed. (Table 3) Instead, mixed type was more frequent in CE patients (34.5%) than LAA (0%) and undetermined (21.1%) (p = 0.037). (Table 3)

**Table 3:** Frequencies of clot types by the stroke subtypes in all retrieved clots.

Stroke subtype	Clot types		
	RBC-rich (n = 13)	Mixed (n = 24)	Fibrin/platelets-rich (n = 56)
LAA (n = 16)	3(18.7%)	0 (0%)	13 (81.3%)
CE (n = 58)	9 (15.5%)	20 (34.5%)	29 (50%)
UD (n = 19)	1 (5.3%)	4 (21.1%)	14 73.7%)

**Fig 3:**
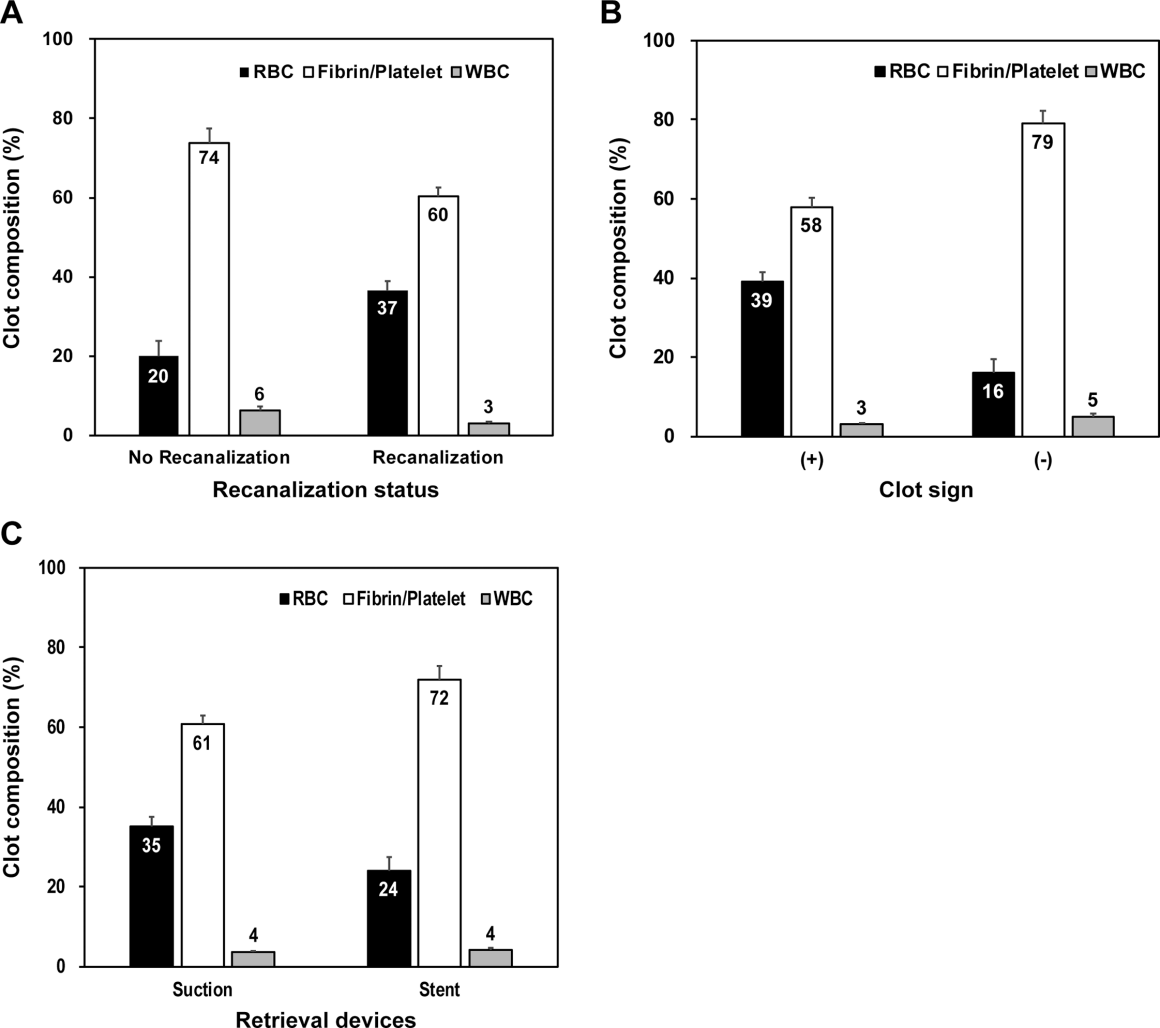
Comparison of clot composition (red blood cells [RBCs], fibrin/platelets, white blood cells [WBCs]) according to recanalization status (A), the presence of clot signs (hyperdense MCA sign on initial computed tomography and/or blooming artifact on initial gradient-echo magnetic resonance images) (B), and the retrieval devices (C).

The total clot area occupied by RBCs was significantly higher in patients who had achieved successful recanalization (37%) than in those who had not (20%, p = 0.001) (Fig 3A). On initial CT or MRI, 25 (67.6%) of 37 patients exhibited clot signs (hyperdense MCA only, n = 1; blooming artifact only, n = 12; both, n = 12). The total clot area occupied by RBCs was significantly higher in patients with clot signs (39%) than in patients without clot signs (16%, p = 0.001) (Fig 3B). The RBC (IVT/IAT, 31.2%; IAT-only, 34.1%, p = 0.561) and fibrin/platelets area (IVT/IAT, 63.8%; IAT only, 62.8%, p = 0.806) was similar regardless of whether IVT was executed before IAT or not. WBC (IVT/IAT, 4.6%; IAT-only, 3.1%, p = 0.018) was only higher in the clots retrieved from patients who received primary IVT. The RBC area was higher in clots retrieved by suction devices (35.6%) than those retrieved by stents (24.1%, p = 0.02). The opposite was true for fibrin/platelet compositions (suction, 60.7%; stent retrieval device, 71.5%, p = 0.02) (Fig 3C).

## Discussion

The results of the present study demonstrated that high RBC composition in clots retrieved during IAT was associated with successful recanalization of occluded vessels, cardioembolic stroke, and clot signs on initial brain images. Thus, our findings may provide insight into the histologic mechanisms underlying recanalization success achieved during IAT.

Clots retrieved from patients with stroke are comprised of RBCs, fibrin/platelets, and WBCs. However, whether RBCs or fibrin/platelets represent the dominant component of clots—as well as the ratio of the two main components—remains controversial. Previous studies have reported RBC values ranging from 32% [22] to 57% [12] in clots obtained from patients who have achieved successful reperfusion. Furthermore, while some previous studies have indicated that RBC composition is lower in patients with cardioembolic stroke than in those with other stroke types [13,21], others have reported contrasting results [9].

Such discrepancies of the previous as well as the present study may have been associated with restrictions in the number patients and the manner in which successful reperfusion was defined. In the present and previous studies, an average of two to three clot retrieval trials was required to attain the final reperfusion status [13,22]. Our findings indicated that clots were retrieved in two-thirds of the patients, although successful recanalization occurred in only half of all retrieval trials. These findings suggest that restriction to one clot per patient is insufficient for evaluating the association between clot composition and retrieval/recanalization. In the present study, all clots obtained after separate retrieval trials were used to evaluate the association between clot composition and radiological findings. Thus, our results provide an accurate representation of this association, as well as the association between clinical characteristics and clot composition.

The variations in clot composition observed among previous studies may also be due to the lack of standardized methods for measuring levels of each component. Indeed, previous studies have utilized H&E staining as well as immunohistochemical and/or component-specific stains for detecting RBCs, fibrin, platelets, and WBCs [11,21,23]. However, reaction bias induced by specimen fixation, tissue processing, antigen retrieval and detection system as well as interpretation bias caused by the selection of antibody panels, sensitivity of the chosen panel, choice of antibody types and clones, results and literature interpretation [24, 25] may influence the measurement of clot composition for immunohistochemically stained clots. One previous study utilized lattice quantification to determine RBC and fibrin/platelet composition in selected areas, although these methods were not used to measure composition based on area within the total clot image [12]. Although semi-automated quantitative and qualitative methods using PhotoShop and ImageJ have also been utilized to measure the RBC, fibrin/platelet, and WBC composition of whole clots [11,13,23,24,26], researchers have utilized different parameters for color, purity, and intensity during analysis.

In the present study, we used the Positive Pixel Count algorithm of the ImageScope program to simultaneously measure the area occupied by RBCs, fibrin/platelets, and WBCs within an entire clot stained using H&E. To precisely measure each composition from the entire clots, we fixed the hue value (0.05), hue width (0.04), saturation (0.04), the intensity of weak pixels (215 in the range from 0 [dark] to 255 [bright white]), to exclude non-stained areas, and the intensity of strong pixels (100) to discriminate RBCs. We only adjusted pixel intensity within a specified range (135–180) to ensure clear discrimination of RBCs and fibrin/platelets. Although the fixing and adjusting of the parameters in ImageScope may differ from Photoshop and ImageJ, the parameter settings utilized in the present study may aid in the development of standardized values to measure clot compositions in H&E-stained clot images.

Despite our findings, there are several issues that remain to be resolved to improve the evaluation of associations between clot composition and clinical status. First, standardization of color/intensity settings for the analysis of digitized clot images is required. The standardization of hue, saturation, and intensity values for H&E-stained clot images may aid in minimizing differences in results obtained using Photoshop, ImageJ, and ImageScope. In addition, further studies are required in order to clarify the clinical significance of the association between high RBC composition and successful recanalization during IAT interventions. One *in vivo* study has suggested that clots rich in RBCs exhibit increases viscosity and elasticity relative to other clots [27]. Additional research has suggested that the physical characteristics of RBC-rich clots are associated with clot retrieval during IAT interventions [28]. Despite these findings, future studies should utilize other mechanical and physical characteristics to verify such an association in patients with acute ischemic stroke. Furthermore, to understand the exact composition of the clot causing arterial occlusion, we need to know whether the retrieved clot is captured from the exact occlusion site, or from a proximal or distal portion of the clot during IAT. Unfortunately, no useful tool to mark or stain the clot causing the occlusion has been developed so far. In the future, the development of marking for the occluded clots might be helpful in understanding the compositional characteristics of the retrieved clots. Finally, a prospective collection of retrieved clots accompanied by clinical and radiological data (e.g., blood pressure and the presence of collateral circulation) are required. Previous research has indicated that the blood pressure gradient across clots is associated with the success of recanalization in patients with poor collateral development [8,28]. However, the other clinical and radiological factors related with clot characteristic have not been well known. yet. In particular, the exact location of the retrieved clots from an occluded vessel is not easy to estimate due to absence of a method to visualize the occlusion site. In the present study, although we collected individual clots from separate catheterizations performed in individual patients, we did not evaluate clinical and radiological data before and after individual clot collection. And, we also could not measure the clot characteristics representing all clots collected from each patient. Thus, future studies should obtain clinical and radiological data both prior to and following individual clot retrieval, as this may aid in elucidating the association among hemodynamic factors, clot characteristics, and successful recanalization. And, the studies should also obtain clot properties representing the clinical and radiological characteristics of all clots retrieved from a patient, also.

The present study demonstrated that high RBC composition in retrieved clots was associated with successful recanalization during IAT. Moreover, high RBC composition was more frequent in patients with cardioembolic stroke, and significantly associated with clot signs observed on initial brain images. Although our parameters for image analysis may have increased the accuracy of our measurements regarding clot composition, further studies are required to evaluate the association between the hemodynamic and physical characteristics of retrieved clots and recanalization success.

## Supporting information

S1 Fig Clot images digitized with different hue width of 0.33, 0.4, and 0.5 at hue value 0.05. (TIF)

S2 Fig Clot images digitized with different saturation values from 0.01 to 0.1 in 6 different clots having compositional differences of RBC and fibrin/platelet. H&E, hematoxylin and eosin stain.
(TIF)

S3 Fig Compositional changes of white blood cell (WBC) in clot images digitized with different saturation values from 0.01 to 0.1 in 6 clots having different compositions of red blood cell (RBC) and fibrin/platelet. (TIF)

S4 Fig Compositional changes of red blood cell (RBC, A) and fibrin/platelet in clot images digitized with different intensity values for weak positive threshold (I_wp_) from 211 to 220 in 6 clots having different compositions of RBC and fibrin/platelets. (TIF)

S5 Fig Compositional changes of red blood cell (RBC), fibrin/platelet (F/P), and white blood cell (WBC) in clot images digitized with different intensity values for positive threshold (I_p_) from 135 to 180 in 6 clots having different compositions of RBC and fibrin/platelet. (TIF)

S1 Table Clinical, radiological and clot compositional data of all included patients. (XLSX)
