# Supplementary figures and images for: High red blood cell composition in clots is associated with successful recanalization during intra-arterial thrombectomy
Source: PLoS One. 2018 May 21;13(5):e0197492. doi: 10.1371/journal.pone.0197492 (PMC5962078; doi:10.1371/journal.pone.0197492)

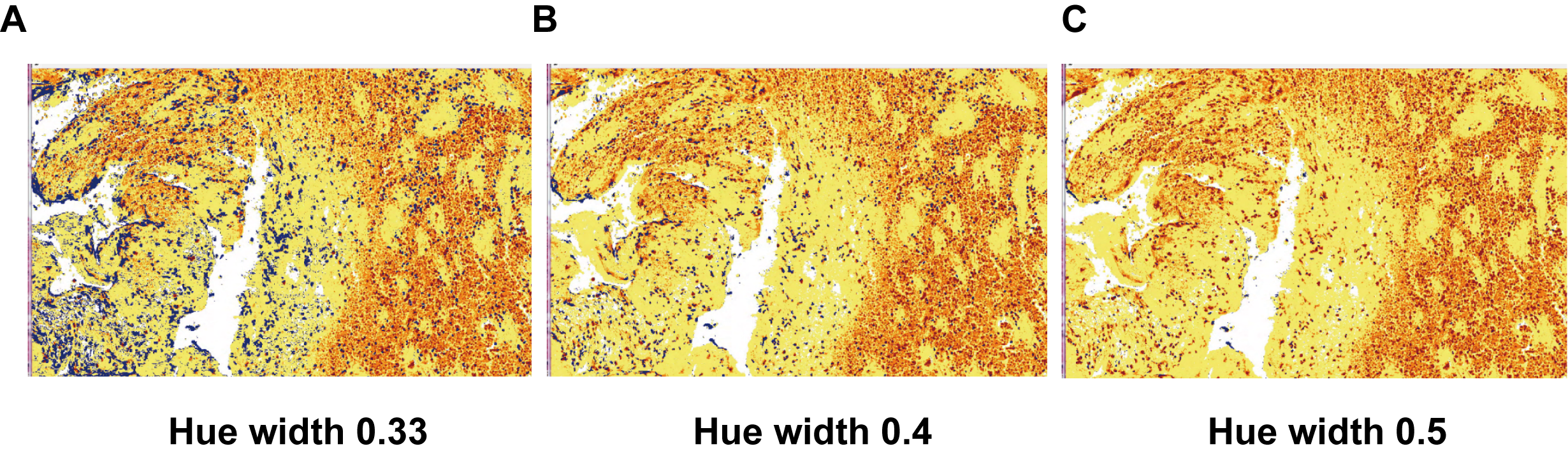

Supplement: S1 Fig — (TIF) [file pone.0197492.s001.tif]

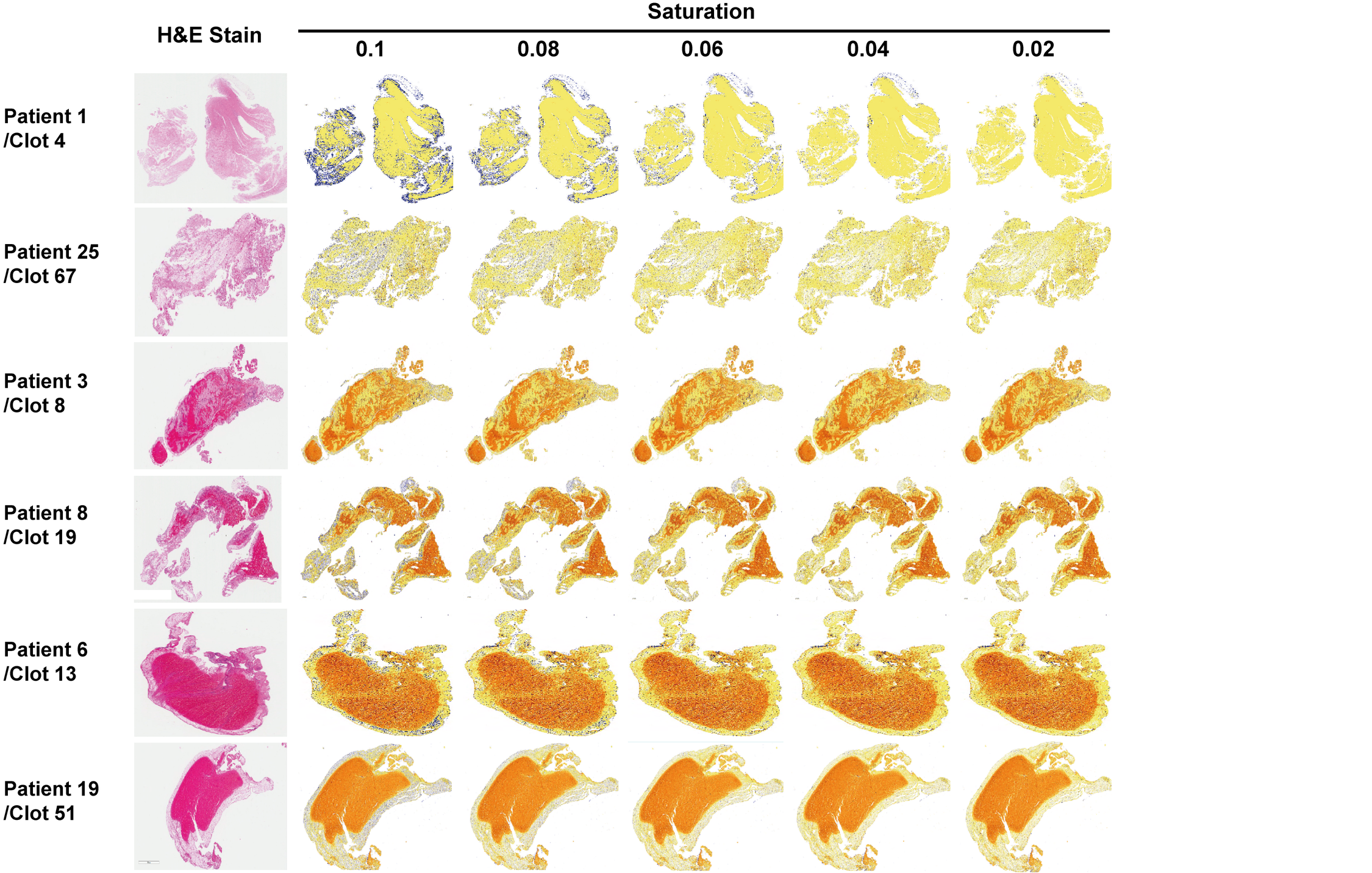

Supplement: S2 Fig — H&E, hematoxylin and eosin stain. (TIF) [file pone.0197492.s002.tif]

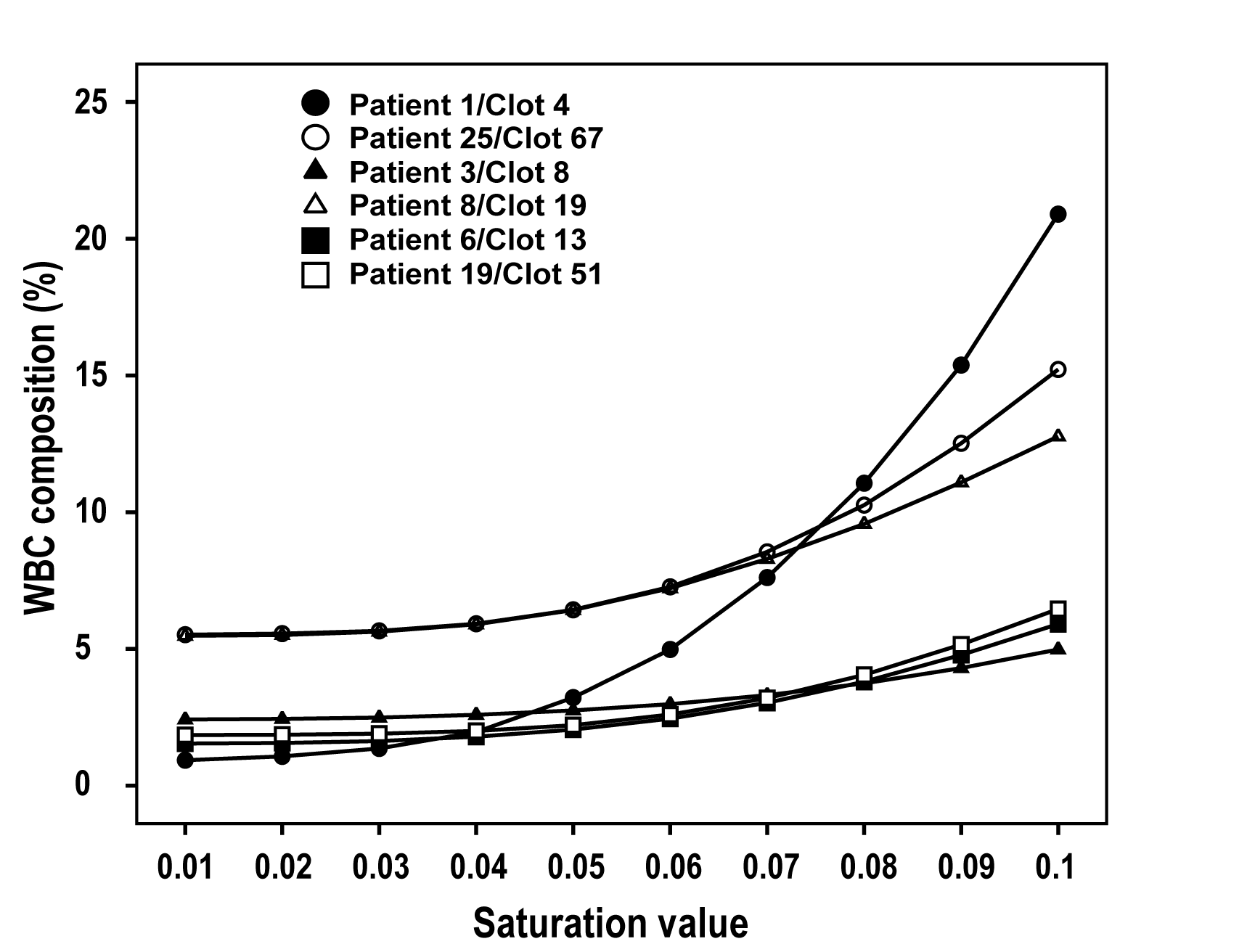

Supplement: S3 Fig — (TIF) [file pone.0197492.s003.tif]

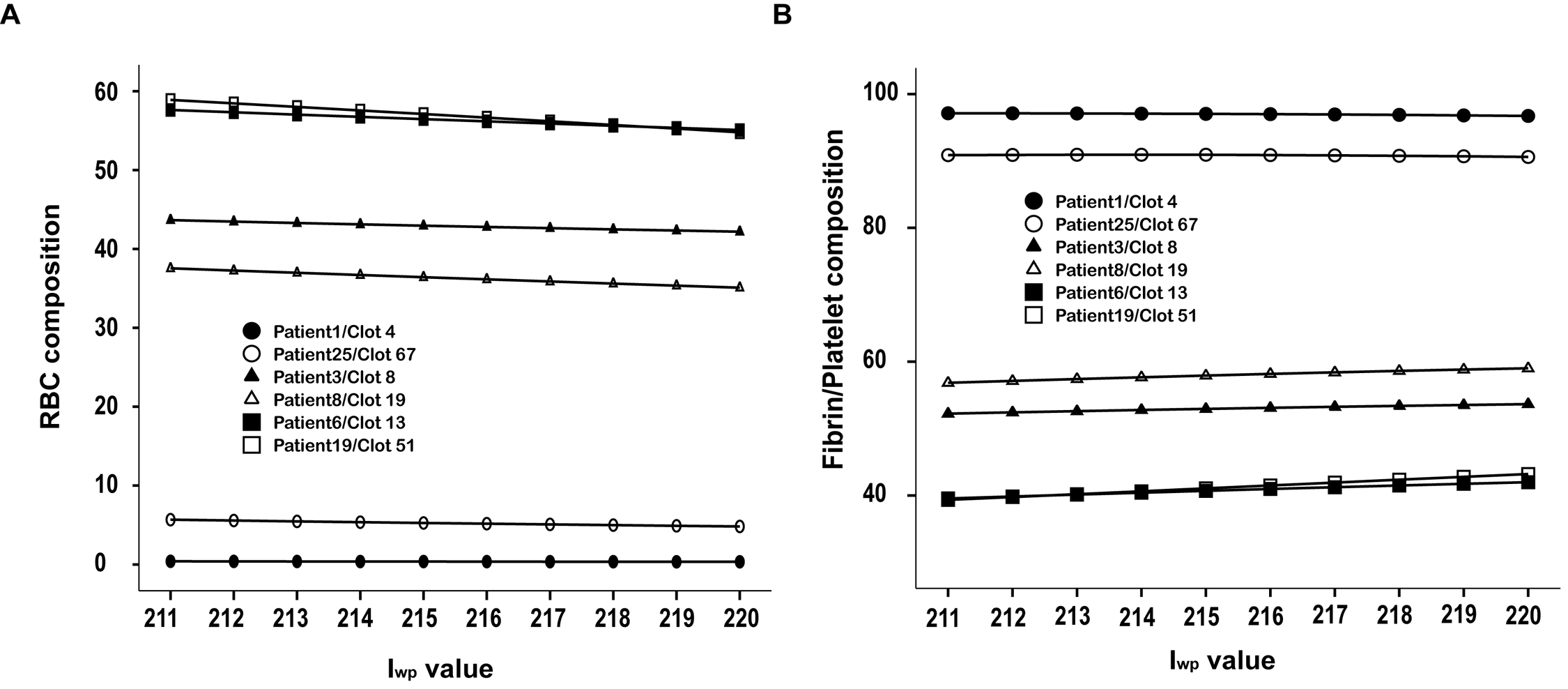

Supplement: S4 Fig — (TIF) [file pone.0197492.s004.tif]

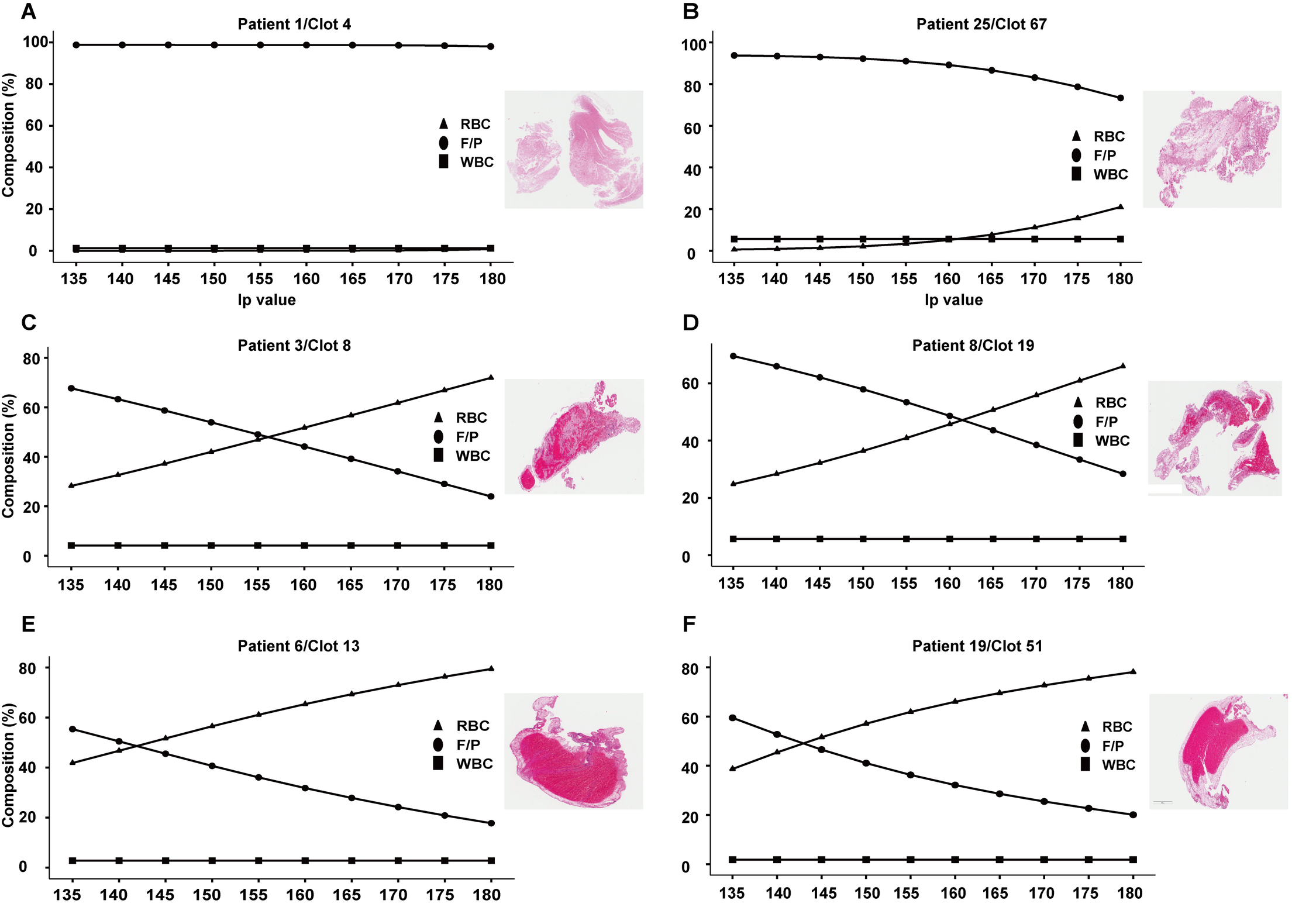

Supplement: S5 Fig — (TIF) [file pone.0197492.s005.tif]
